# *In Vitro* Tests for Assessing the Neutralizing Ability of Snake Antivenoms: Toward the 3Rs Principles

**DOI:** 10.3389/fimmu.2020.617429

**Published:** 2021-01-11

**Authors:** José María Gutiérrez, Mariángela Vargas, Álvaro Segura, María Herrera, Mauren Villalta, Gabriela Solano, Andrés Sánchez, Cristina Herrera, Guillermo León

**Affiliations:** ^1^ Instituto Clodomiro Picado, Facultad de Microbiología, San José, Costa Rica; ^2^ Facultad de Farmacia, Universidad de Costa Rica, San José, Costa Rica

**Keywords:** neutralization, lethality assays, *in vitro* assays, analgesia, 3Rs, antivenoms, snake venoms

## Abstract

There is an urgent need to strengthen the implementation of the 3Rs principle (Replacement, Reduction and Refinement) in the use of experimental animals in toxinological research and in the assessment of the neutralizing efficacy of snake antivenoms. This is a challenging task owing to the inherent complexity of snake venoms. The state of the art on this topic is hereby reviewed, with emphasis on the studies in which a correlation has been observed between *in vivo* toxicity tests and *in vitro* surrogate assays, particularly in the study of lethal activity of venoms and its neutralization. Correlations have been described with some venoms-antivenoms when using: (a) enzyme immunoassays, (b) hemagglutination, (c) enzyme assays (proteinase, phospholipase A_2_), (d) *in vitro* coagulant effect on plasma, (e) cell culture assays for cytotoxicity, (f) functional assays for assessing neurotoxicity *in vitro*, (g) use of hens’ eggs, and (h) antivenomics. Additionally, the routine introduction of analgesia in these assays and the design of more ‘humane’ protocols for the lethality test are being pursued. It is expected that the next years will witness a growing awareness of the relevance of the 3Rs principles in antivenom testing, and that new *in vitro* alternatives and more ‘humane’ experimental designs will emerge in this field.

## Introduction

Snakebite envenoming exerts a heavy toll in terms of mortality and disabilities on a global basis (1). Owing to their public health relevance, the World Health Organization (WHO) included these envenomings as a category A disease in its list of Neglected Tropical Diseases in 2017 (2), and a resolution on the subject was adopted at the World Health Assembly in 2018 (3). More recently, the WHO launched a global strategy to prevent and control these envenomings, aimed at reducing by 50% the number of deaths and amputations due to this disease by the year 2030 (4). This strategy is based on four pillars, one of which is to ‘ensure safe, effective treatment’.

The centerpiece in the therapy of snakebite envenomings is the timely administration of safe and effective antivenoms, which are preparations of IgGs or IgG fragments prepared from the plasma of horses or other animals immunized with venoms of one snake species (monospecific antivenoms) or several species (polyspecific antivenoms) (5). Upon parenteral administration in envenomed patients, antivenom antibodies bind to venom components in the circulation or in tissue compartments and contribute to their elimination. Generally, antivenom therapy is complemented by ancillary treatments which vary depending on the pathophysiology of envenomings (1). Antivenom efficacy is evaluated at the preclinical level by assessing its capacity to neutralize the lethal action of venoms in animal models, usually mice (5, 6). This is the gold standard of antivenom efficacy which is required before antivenoms are introduced into clinical use and as part of the routine quality control of antivenoms by manufacturers and regulatory agencies. The basic protocol for these neutralization assays involves the incubation of venom and antivenom prior to administration in animals. Another experimental option, which is not routinely used in quality control laboratories but which better mimics the actual circumstances of a snake bite, is the rescue-type assay, in which venom is injected first and antivenom is administered afterwards. In addition to lethality, depending on the toxicity profile of venoms, the assessment of neutralization of other toxic activities is also recommended, such as hemorrhagic, myotoxic, dermonecrotic, defibrinogenating, and *in vitro* coagulant activities, depending on the venom (5, 6). Except for the *in vitro* coagulant activity, the rest of these assays involve the use of high numbers of mice, with the consequent suffering and distress inflicted in these animals because of the toxic action of venoms.

There is a growing awareness on the need to significantly reduce the number of mice used in antivenom assessment, as well as the pain and distress involved in these tests, along the philosophy of the 3Rs (Replacement, Reduction and Refinement) proposed by Russell and Burch (7). A significant amount of work has been devoted by many groups to the search of *in vitro* alternatives to these animal tests, and to the refinement of these assays. Owing to the high variability of snake venom composition and mechanisms of action, no simple generalizations can be made regarding the implementation of these alternative tests. However, there are examples of *in vitro* assays which show a good correlation with the *in vivo* tests, and further work is urgently needed in this field. The present review presents the state of the art in the development of *in vitro* tests for antivenom preclinical efficacy assessment. The review focuses mostly on studies in which the correlation between *in vitro* and *in vivo* tests was evaluated.

## The Challenge of Finding Suitable *In Vitro* Tests for Assessing Antivenom Efficacy

One of the main challenges for finding suitable *in vitro* tests that would substitute *in vivo* experiments in the evaluation of antivenoms has to do with the complexity of snake venoms and snakebite envenomings. In some cases, the toxic profile of venoms depends on the action of one or few toxins which induce a single toxicological effect, e.g., the action of some neurotoxic elapid venoms which act by blocking the neuromuscular junctions. Thus, once these components are identified, it is feasible to develop immunochemical or functional *in vitro* tests to study the ability of antivenoms to react and neutralize these venoms. However, for many snake venoms this is not the case, as the overall pathophysiology of envenoming is the result of the combined action of several toxins acting on different tissues or physiological systems (1), a fact that complicates the development of *in vitro* surrogate tests. Toxins may act synergistically or additively (8) and have complex toxicokinetic and toxicodynamic profiles which play a role in the *in vivo* assays. Moreover, effects such as cardiovascular or renal alterations, as well as local tissue damage, involve multifactorial processes difficult to reproduce *in vitro*.

In addition, there is a growing body of evidence indicating that the pathophysiology of many envenomings derives not only from the direct action of toxins on tissues, but also from endogenous processes in the organism, such as inflammatory cascades resultant of the action of toxins or the generation of damage-associated molecular patterns (DAMPs) from affected tissues, which contribute to the pathophysiological alterations (9, 10). Thus, the study of snake venom composition and mechanisms of action, and the identification of the main toxins responsible for the predominant toxicological effects provide relevant information for the knowledge-based design of alternative *in vitro* assays that correlate with *in vivo* toxicity tests.

## Assessment of Antivenom Neutralizing Efficacy at Different Stages During the Manufacturing Process

The quality control of antivenoms, in terms of assessing their neutralizing efficacy against medically-relevant snake venoms, is generally carried out at two stages: (a) in-process, i.e. along the plasma fractionation procedures for generating purified IgG or F(ab’)_2_ preparations, and (b) in the final product, before the antivenom is released for medical use in the health systems. The in-process quality control is carried out by the manufacturer, whereas the quality control of the final product is done by the manufacturer and, in some countries, by the national regulatory agencies as well.

Generally, the final quality control of antivenoms necessarily involves the test for the neutralization of lethal activity of venoms in mice, which is the gold standard for antivenom efficacy assessment (5). On the other hand, the in-process quality control of antivenom efficacy offers opportunities for the implementation of *in vitro* tests aimed at detecting whether there is a loss of neutralizing antibodies during plasma fractionation. However, many manufacturing laboratories routinely use the mouse lethality assay for these in-process quality control analyses. It is necessary to develop *in vitro* assays which correlate with the *in vivo* tests for the in-process quality control of antivenoms. This will greatly reduce the number of mice utilized during the manufacturing process. Likewise, the follow up of the development of neutralizing antibody titers in the plasma of horses along the immunization scheme, in order to establish the best time for starting the bleeding protocols, could be done by using *in vitro* tests that offer a good correlation with the *in vivo* potency assays, hence reducing the need for the latter.

## The Origins of *In Vitro* Testing of Antivenoms

Since the dawn of antiserum therapy for snakebite envenomings, the assessment of the neutralizing potency of antivenom was based on the ability to abrogate the lethal action of venoms in various animal models (11, 12). In addition, even at those early times of antivenom development, scientists were searching for *in vitro* tests for assessing antivenom efficacy. Albert Calmette, one of the founders of snake antivenom therapy, described the parallelism between neurotoxicity and indirect hemolysis in neurotoxic (elapid) venoms, and between hemorrhagic activity and proteolysis in viperid venoms (11). Based on such parallelism, he developed laboratory assays to assess the neutralization of hemolytic and proteolytic activities of venoms by antivenoms and described the relationship with the neutralization of *in vivo* toxicity (11). In his book of 1907 *Les Venins, les Animaux Venimeux et la Sérotérapie Antivenimeuse*, referring to these *in vitro* methods, he states (page 269) ‘These various control methods make it possible to verify exactly the activity of *sérums antivenimeux* without it being necessary to use animal testing’ (11). Likewise, Vital Brazil, working in São Paulo, Brazil, described experiments on the neutralization of *in vitro* coagulant and proteolytic activities of venoms, and on the formation of precipitates when venoms and antivenoms were allowed to react in a test tube (12).

Ahuja and Brooks (13) described an *in vitro* hemolysis test for assessing the neutralizing potency of cobra antivenom in India, which correlated with the neutralization of lethality. In South Africa, Paul A. Christensen studied several *in vitro* activities of venoms (hemolysis, rennin-like effect, gelatinase and anticoagulant activities) and their neutralization by antivenoms. He found no correlation between the neutralization of lethality and *in vitro* hemolysis in the case of *Naja flava* (now *Naja nivea*) venom (14). As will be described later, no generalizations can be made regarding the possible substitution of *in vivo* toxicity tests by *in vitro* assays, owing to the great variability in the composition and action of snake venoms.

## Enzyme Immunoassays

Theakston et al. (15) introduced the enzyme-linked immunosorbent assays (ELISAs) for the quantification of venom and antivenom. An ELISA was then used to quantify antivenom antibodies in several commercial antivenoms used in Africa and some rabbit experimental antivenoms and this was correlated with the neutralization of lethality in mice (16). A good correlation was described when using the venoms of the African species *Bitis arietans*, *Echis carinatus* (now *E. ocellatus*), *Naja haje* and *N. nigricollis*. Similar descriptions of significant correlation between ELISAs and *in vivo* neutralization of lethality have been described for a monospecific *Naja naja kaouthia* antivenom (17), monospecific *Crotalus durisus terrificus* antivenom (18), bispecific *Bothrops alternatus* and *B. pubescens* antivenom (19), and monospecific *Daboia siamensis* antivenom (20).

In contrast, poor correlation between ELISA and neutralization of lethality was described for the bothropic antivenom manufactured in Brazil when tested against the venom of *Bothrops jararaca* (18, 21) and a monospecific *Micrurus nigrocinctus* antivenom toward its homologous venom (22). Hence, the feasibility of using ELISAs for assessing antivenom potency must be made on a case by case basis. An explanation for the lack of correlation in the case of some venoms and antivenoms is that proteins that do not play a role in toxicity may be highly immunogenic and, therefore, the immune response detected by ELISA may reflect antibody titers against toxicologically irrelevant components. This is illustrated in the case of the venom of the black mamba *Dendroaspis polylepis*, whereby antivenoms show highest antibody titers against high molecular mass non-toxic metalloproteinases, whereas titers against neurotoxins are lower (23).

A solution to this situation is the identification and isolation of venom components having the highest toxicity in a venom, by assessing the ‘toxicity score’ of venom fractions (24). Once these toxins are identified, ELISAs can be developed for the quantification of antibodies against them. This increases the likelihood of correlation between immunoassays and the *in vivo* neutralization of lethality. This concept has been proven in the case of antivenom against *Naja naja siamensis*, since a higher correlation was observed when immunoassays were carried out using a purified α-neurotoxin, as compared to crude venom (17). Similarly, a higher correlation was described for the Brazilian bothropic antivenom when using a hemorrhagic fraction of the venom of *B. jararaca* as compared to crude venom, but not when using a phospholipase A_2_ (PLA_2_)-rich fraction (21, 25). The growing body of information of snake venom proteomes, together with the identification of key toxins, provides valuable evidence for the setting of these more directed ELISAs.

In the cases of venoms whose predominant toxins represent a high percentage of venom composition, ELISAs using crude venoms are likely to give a good correlation with *in vivo* toxicity tests. This is the case of the venom of the South American rattlesnake *C. d. terrificus*, in which the potent neurotoxin crotoxin comprises 60% of the venom (26). Similarly, the venom of the cobra *Naja kaouthia* has a high concentration of α-neurotoxins which display the highest toxicity score (27). It is necessary to explore medically relevant venoms and their corresponding antivenoms to establish in which cases good correlation between ELISA and neutralization of lethality can be achieved by using crude venoms or when it is recommended to use purified toxins.

## Passive Hemagglutination and Hemagglutination Inhibition

A method based on passive hemagglutination and its inhibition was developed for testing a monospecific *Naja naja siamensis* antivenom using glutaraldehyde treated sheep erythrocytes coupled with toxin 3, a neurotoxin from this venom (28). A similar method was used by Pradhan et al. (29) to assess whether it correlates with the *in vivo* neutralization of lethality. Erythrocytes treated with glutaraldehyde and then with tannic acid were coupled with *Naja naja* venom and then incubated with varying dilutions of the antivenom. Also, inhibition of hemagglutination was carried out by incubating antivenom with venom, followed by addition to venom-coated erythrocytes. A good correlation between these tests and the *in vivo* neutralization of lethality was observed. It remains to be seen whether this method works only for these α-neurotoxin-rich venoms or also for other venoms having a different toxin composition.

## Neutralization of *In Vitro* Enzymatic Activities

Snake venoms are rich in hydrolytic enzymes. The proteomic analyses of viperid venoms have revealed a predominance of snake venom metalloproteinases (SVMPs), phospholipases A_2_ (PLA_2_s) and serine proteinases (SPs), with variations between and within species (1). In turn, elapid venoms are generally rich in PLA_2_s (1). These enzymes are responsible for some of the main pathophysiological effects in envenomings. SVMPs induce hemorrhage and coagulopathies (30, 31), PLA_2_s are responsible for muscle necrosis and neurotoxicity, depending on the enzyme (32, 33), and serine proteinases induce defibrinogenation and hypotension (31, 34). Therefore, the study of *in vitro* activities associated with these enzymes has been pursued and correlated with *in vivo* toxicity.

Several studies have demonstrated a correlation between the neutralization of *in vitro* coagulant activity of venoms, associated with the action of procoagulant SVMPs and serine proteinases, and neutralization of lethality. This was described for *Calloselasma rhodostoma* venom and a monospecific antivenom (35) by using sheep plasma (for assessing *in vitro* coagulation) and intraperitoneal injection in mice (for assessing lethality). Similar findings were reported in the case of *Bothrops jararaca* venom and the Brazilian bothropic antivenom, whereby *in vitro* coagulant activity was assessed by rotational thromboelastometry (ROTEM) on chicken plasma, and lethality was studied in mice by the i.p. route (36). A correlation was also described between neutralization of *in vitro* coagulant activity and neutralization of lethality (i.p. route) in the case of *Bothrops asper* venom and a polyspecific Costa Rican antivenom (37).

To further expand these observations, we have assessed the correlation of these activities based on data included in two publications which evaluated many antivenoms. In a study carried out in Latin America in which seven polyspecific viperid antivenoms were assessed against venoms of six species of *Bothrops* sp. (38), a significant correlation was found (R = 0.492, p = 0.0011, n = 41). Two publications evaluated the neutralization of these effects by seven antivenoms against venoms of *Echis ocellatus* from various locations in sub-Saharan Africa (39, 40). A significant correlation between neutralization of lethality and *in vitro* coagulant activity was observed (R = 0.7643, p = 0.0009, n = 15). These findings support the view that the study of neutralization of *in vitro* coagulant activity could be a surrogate test for estimating the neutralizing ability of viperid antivenoms. Additional studies with other venoms and antivenoms are required to further substantiate this correlation. It is necessary to standardize the conditions of the *in vitro* coagulant assay, including the type of plasma used and the assessment of clot formation. In order to standardize the performance of this *in vitro* test in quality control laboratories, it is recommended that reference antivenoms be prepared and run in parallel every time an antivenom is being evaluated for its efficacy.

Other studies have shown correlation between neutralization by antivenoms of PLA_2_ activity *in vitro* and neutralization of lethality in mice in the cases of venoms of *Bothrops asper* (41), *Crotalus durissus terrificus* (42), and *Micrurus nigrocinctus* (22), using simple indirect hemolytic assays for the determination of PLA_2_ activity. Further studies are necessary to assess whether these *in vitro* enzymatic assays correlate with lethality in a larger number of venoms and antivenoms. There are venoms in which the main toxicity is due to presynaptically-acting neurotoxic PLA_2_s (43). Such are the cases of *Oxyuranus scutellatus*, *Crotalus durissus*, and *Bungarus* sp venoms, characterized by the presence of the potent PLA_2_ neurotoxins taipoxin, crotoxin and bungarotoxin, respectively (44). It is likely that the neutralization by antivenoms of PLA_2_ activity *in vitro* of these venoms or purified β-neurotoxins correlates with the neutralization of lethality. Owing to the simplicity and low cost of these *in vitro* assays, they could be highly convenient for introduction in antivenom manufacturing laboratories to assess the development of immune response in horses and for in-process analysis of the neutralizing potency of antivenoms, with the consequent reduction in the number of mice.

## Surrogate Tests for The Study of Neutralization of Other Toxic Activities

**Figure 1:**
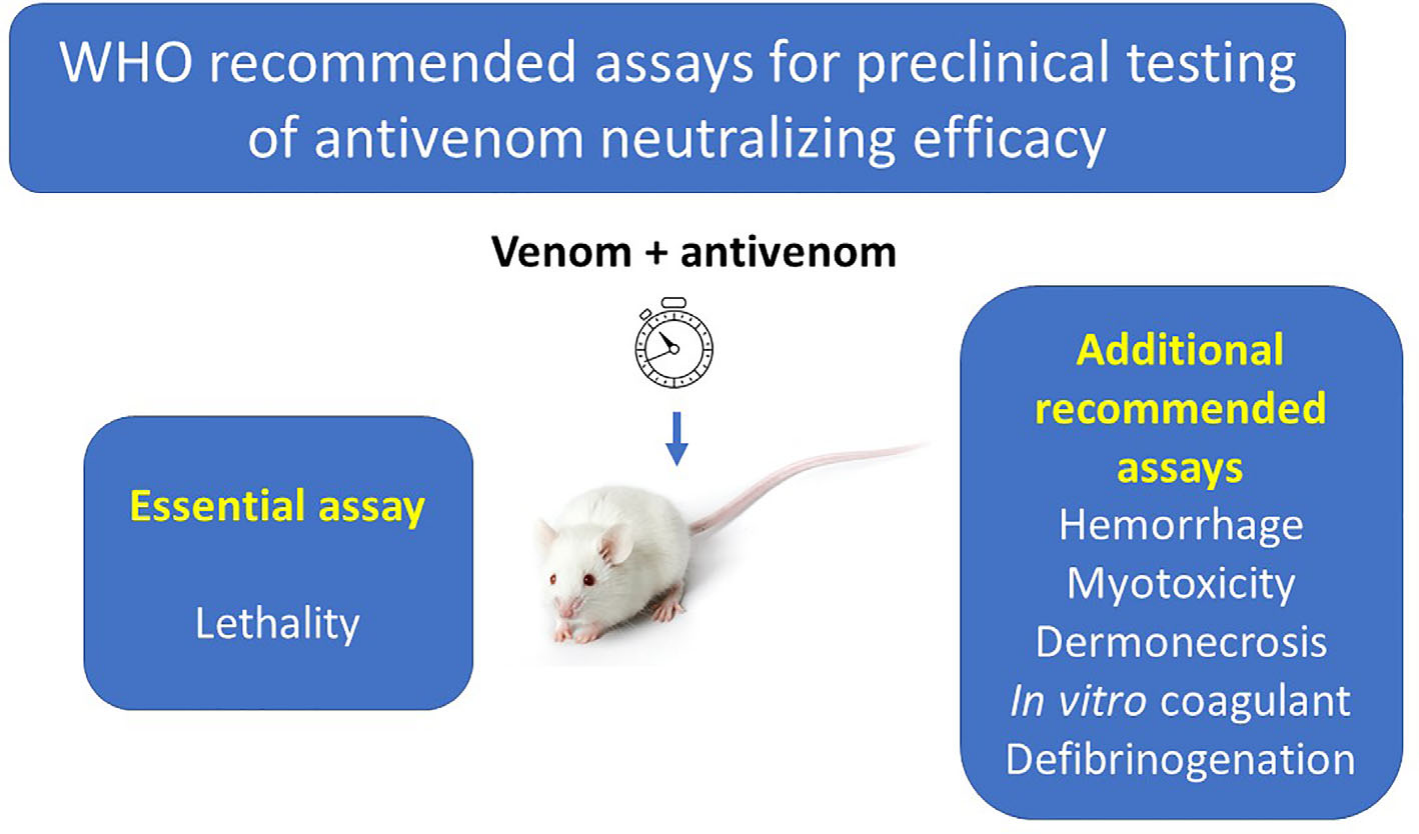
Assays included in the WHO Guidelines for the Production, Control and Regulation of Snake Antivenom Immunoglobulins for the assessment of antivenom preclinical efficacy. The WHO divides these assays into the ‘essential assay’, which is the analysis of the neutralization of lethal activity of venoms, and ‘additional recommended assays’, which assess the neutralization of other toxic activities, depending on the toxicological profile of the venom under study.

The complexity of the pathophysiology of snakebite envenomings calls for a more comprehensive evaluation of the neutralizing ability of antivenoms, involving not only the neutralization of lethality, the gold standard of preclinical antivenom efficacy, but also of hemorrhagic, defibrinogenating, myotoxic and dermonecrotic activities, which play key roles in envenomings by diverse snake species (6). The WHO has established the neutralization of lethality as the ‘essential’ test for the preclinical evaluation of antivenoms and, depending on the venom, additional ‘supplementary’ tests are recommended when new antivenoms are developed or when an existing antivenom is being distributed to a new geographic region (5) (**Figure 1**). For example, in the case of most viperid venoms, neutralizations of hemorrhagic, myotoxic, and defibrinogenating activities are recommended. Likewise, assessment of antivenoms against venoms of necrotizing spitting cobras (*Naja* sp.) should include the neutralization of dermonecrotic activity (6). Since these supplementary tests involve the use of mice, the search for alternative *in vitro* assays is necessary.

Hemorrhagic activity by viperid venoms is predominantly due to the action of SVMPs on the basement membrane that surrounds and provides support to endothelial cells in capillary blood vessels. In particular, the hydrolysis of type IV collagen is critical for microvessel disruption (30, 45, 46). Inhibition of venom metalloproteinase activity by chelating agents or peptidomimetic inhibitors results in the abrogation of hemorrhage (47–49). Hence, *in vitro* inhibition of proteinase activity of venoms may constitute a surrogate alternative for assessing the neutralization of hemorrhagic effect. A significant correlation between neutralization of hemorrhage and of hydrolysis of casein *in vitro* was shown for the polyspecific viperid antivenom manufactured in Costa Rica when tested against ten venoms (50). A higher correlation is expected if physiologically relevant substrates, such as basement membrane components, are used as substrates, a hypothesis to be tested. An ELISA-based assay was developed for the quantification of gelatinase activity of viperid venoms. It is based on the addition of venoms to gelatin-coated wells in plates, followed by incubation. Then, anti-gelatin antibodies are added followed by a conjugate and color development (51). Activity was higher in viperid venoms, as compared to elapid and ‘colubrid’ ones. Activity was abolished by EDTA, indicating that it is due to SVMPs. Whether this assay offers a good correlation with hemorrhagic activity of venoms and its neutralization by antivenoms remains to be determined. Likewise, a high correlation was described between an ELISA using a monoclonal antibody raised against the PIII hemorrhagic SVMP jararhagin and the hemorrhagic activity of individual venoms of *Bothrops jararacussu* (52). This could be the basis of an ELISA aimed at assessing the neutralizing ability of antivenoms against hemorrhagic venoms.

Coagulopathy, i.e. defibrinogenation, is a common consequence of envenomings by viperids and some elapids and ‘colubrids’ and contributes to the systemic hemorrhage characteristic of these envenomings (1, 31, 53). Defibrinogenating effect is tested *in vivo* by determining the minimum dose of venom that renders blood unclottable in experimental animals (54, 55). Defibrinogenation is the consequence of the consumption of clotting factors owing to the action of procoagulant enzymes in venoms, i.e., factor X activators, prothrombin activators and thrombin-like enzymes (31, 56). Therefore, the *in vitro* coagulant activity of venoms is likely to be a surrogate test for *in vivo* defibrinogenating effect. Indeed, a relationship was shown between the ability of a polyspecific antivenom to neutralize *in vitro* coagulant and *in vivo* defibrinogenating activities of five viperid venoms (55).

Myotoxic activity of snake venoms is predominantly due to the direct action of PLA_2_s, and PLA_2_ homologs, on the plasma membrane of muscle fibers (43, 57). However, no correlation between inhibition of PLA_2_ activity and of myotoxicity is expected because in many venoms enzymatic phospholipid degradation is mostly due to non-toxic enzymes, as in the case of *Bothrops asper* which has an acidic PLA_2_ with high enzymatic activity but being devoid of myotoxicity (58). An alternative is the assessment of cytotoxicity on muscle cell lines, i.e., myoblasts and myotubes of the C2C12 line. Myotubes are good models of mature muscle fibers and are highly susceptible to myotoxic PLA_2_s (59). The correlation between neutralization by antivenoms of *in vivo* myotoxicity and *in vitro* cytotoxicity on myotubes must be studied. Likewise, the assessment of cytotoxicity in cell culture systems could become a surrogate assay for the analysis of dermonecrosis, a clinically significant effect of envenomings by spitting cobras in Africa and Asia (1, 53). The myogenic cell line C2C12 was used to assess cytotoxicity by venoms of five species of *Naja* sp. from Africa and its neutralization by a polyspecific antivenom (60), but whether this assay correlates with *in vivo* dermonecrosis remains to be investigated. A cell culture test using human keratinocytes was developed to study the cytotoxic action of *Naja* sp. venoms and its neutralization by recombinant antibodies (61). Since these venoms induce demonecrosis, this *in vitro* test could be of value to assess the neutralizing efficacy of antivenoms. Cytotoxicity on kidney cell lines has been used in the analysis of nephrotoxic effects of venoms and toxins (62) and must be explored as a surrogate test for assessing antivenom efficacy, although venom-induced nephrotoxicity is of a multifactorial pathogenesis which also involves the effects of hemodynamic alterations (63).

## *Ex Vivo* and *In Vitro* Assessment of Neurotoxicity

Neuromuscular paralysis leading to respiratory arrest is one of the predominant effects of snakebite envenomings, particularly those caused by species of the family Elapidae, but also by some species of the family Viperidae (1, 53). It results from the action of a variety of neurotoxins at the neuromuscular junctions. Post-synaptically acting polypeptides of the three finger toxins (3FTx) family (α-neurotoxins) act by binding with high affinity to the cholinergic nicotinic receptor (AChR) at the motor end-plate of muscle fibers (64). Neurotoxicity is also due to the action of PLA_2_s at the nerve terminal (β-neurotoxins), by hydrolyzing phospholipids of the plasma membrane, inducing a calcium influx and the consequent alteration of the neurotransmitter exocytotic machinery (65). Other types of neurotoxins include the dendrotoxins, present in mamba (*Dendroaspis* sp) venom, which are inhibitors of the voltage-dependent potassium channels (66). Neurotoxins play a key role in the lethality of snake venoms.

*Ex vivo* neuromuscular preparations have been used by several groups to study the neurotoxic effect of venoms and isolated toxins. The most often used preparations are the chick biventer-cervicis and the mouse phrenic-diaphragm. Once dissected out, these are placed in a bath containing a physiological solution, and muscle twitches are evoked by electrically stimulating the nerve (67). Neurotoxicity is evidenced by the blockade of evoked muscle contractions. This system has been used to assess the ability of antivenoms to neutralize the neuromuscular blocking effect [see, for example, Barfaraz and Harvey (68); Camargo et al. (69), Silva et al. (70)]. In the majority of these studies, the correlation with neutralization of lethality *in vivo* was not investigated, although it is likely that, owing to the relevance of neuromuscular paralysis in the overall toxicity of these venoms, such correlation is likely to occur. Herrera et al. (71) described a relationship between the neutralization of lethality and *ex vivo* neuromuscular blocking activity of the venom of taipan (*Oxyuranus scutellatus*) by two antivenoms. This system is also useful to assess the myotoxic effect of venoms (67). These tests, however, require a specialized laboratory, and are therefore difficult to adapt to the routine quality control analysis of antivenoms. In addition, being *ex vivo* tests, they involve the use of animals.

An alternative to assess the inhibition of post-synaptically acting α-neurotoxins is an assay that quantifies the binding of these neurotoxins to purified AChR, such as those from the electric organ of fish, such as *Torpedo californica* (72). Non-radioactive variations of this assay have been described, which have great potential for antivenom evaluation *in vitro*. The basic set up of these procedures is based on the binding of purified AChR to α-neurotoxin bound to wells in microplates. After a washing step, antibodies against AChR are added, followed by conjugated secondary antibodies (73, 74). This procedure allows the detection of α-neurotoxins in venoms by a competition step whereby the venom is incubated with AChR before the addition to the α-neurotoxin coated plate (74).

**Figure 2:**
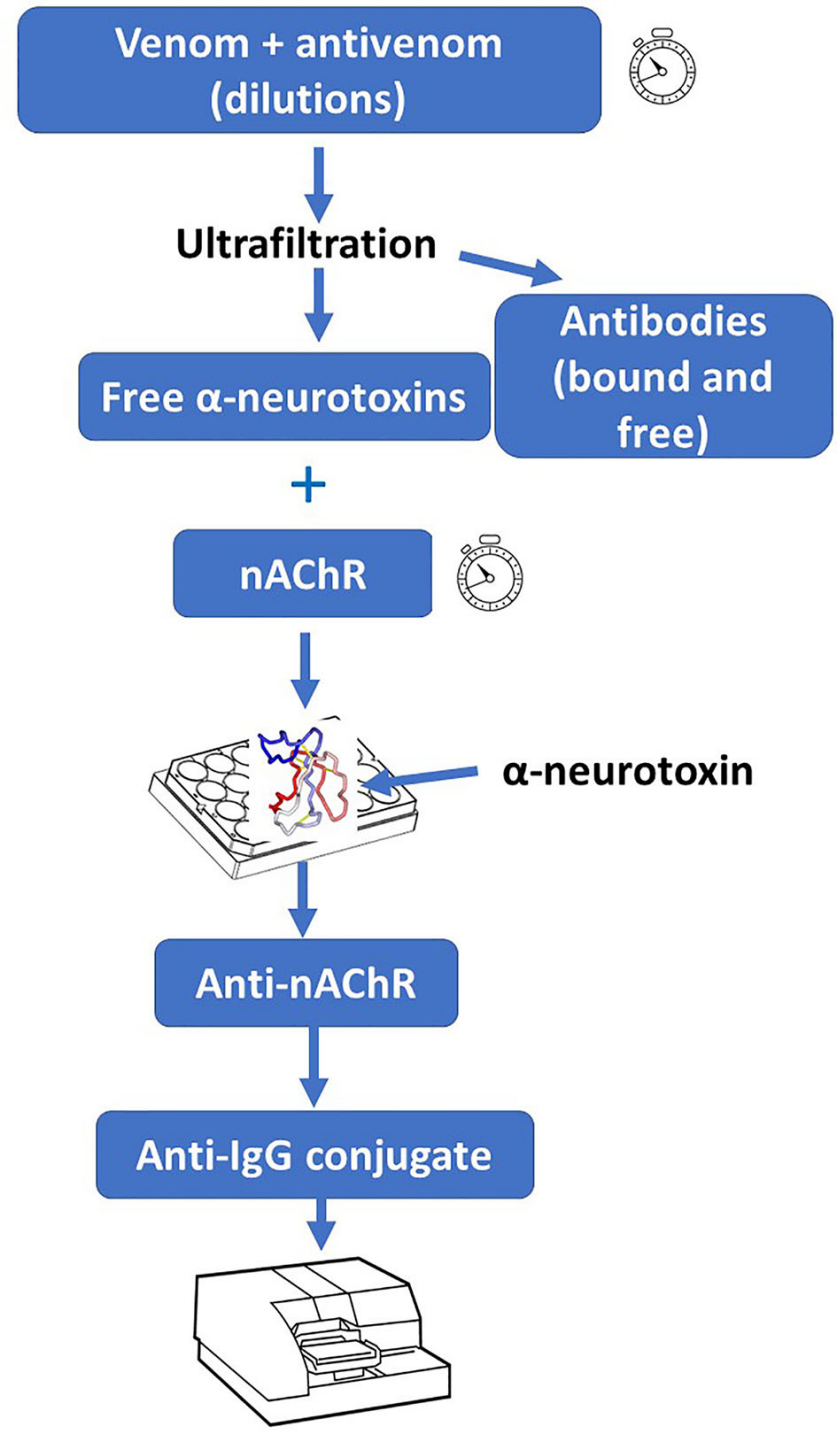
*In vitro* assay for the assessment of the ability of antivenoms to bind to post-synaptically acting α-neurotoxins from snake venoms. A solution containing a fixed concentration of venom is incubated with various dilutions of antivenom. Then, antibodies (both free and venom-bound) are removed from free low molecular mass toxins (including neurotoxins) by ultrafiltration. The filtrate (containing these toxins) is incubated with purified nicotinic acetylcholine receptor (nAChR). Afterwards, the preparation is added to plate wells that had been coated with a purified α-neurotoxin. Upon incubation and washing, anti-nAChR antibodies are added, followed by washing and addition of conjugated anti-IgG antibodies. After adding the corresponding substrate, the absorbance is recorded. The nAChR preparation, which is obtained from the electric organ of fish, could be substituted by synthetic peptides containing the binding site for α-neurotoxins. For details of this procedure, see Ratanabanangkoon et al. (74).

These procedures have been adapted for the study of the ability of antivenoms to bind α-neurotoxins and thus to inhibit their binding to AChR (22). An adaptation of this assay was used to assess its correlation with venom LD_50_ of 20 elapid snake venoms, as well as the correlation of the neutralizing efficacy of an antivenom with the inhibition of AChR binding. In both cases a significant correlation was found, especially in venoms containing a predominance of α-neurotoxins (75). Owing to its simplicity and high-throughput nature, this assay could be adapted to antivenom development and quality control laboratories in the case of elapid neurotoxic venoms rich in α-neurotoxins (**Figure 2**). A potential limitation to the widespread implementation of these assays is the availability of purified AChR. This could be circumvented by the use of mimotopes and peptides derived from AChR which bind to α-neurotoxins (76, 77), as this will avoid the need to obtain the receptor from rays or eels.

For venoms in which β-neurotoxins predominate, a possible *in vitro* alternative would be the neutralization of PLA_2_ enzymatic activity of the purified predominant neurotoxins. Examples are taipoxin in *Oxyuranus scutellatus* (78), β-bungarotoxins in *Bungarus* sp. (79), and crotoxin in *Crotalus durissus* (26) venoms. In the case of venoms such as those of *Bungarus* sp. and *Micrurus* sp., which present both α- and β-neurotoxins, the two assays (AChR binding and PLA_2_ activity) can be used. In the cases of venoms, such as those of *Dendroaspis* sp, rich in other types of neurotoxins, i.e. dendrotoxins (80), an as yet unexplored possibility would be the use of patch-clamp methods using oocytes expressing relevant receptors, such as voltage-dependent potassium channels (81) in the case of dendrotoxins. These, however, require electrophysiology facilities which are not readily available in antivenom quality control laboratories.

## Hen’s Eggs as A Model for Testing Venom Toxicity and Neutralization by Antivenoms

The use of hen’s eggs at a developmental stage when no reflex pain arcs have yet developed was proposed as a model to assess venom toxicity and neutralization by antivenoms (82, 83). Venom solutions are applied to filter paper discs and then placed over the yolk sac membrane of shell-less eggs, followed by incubation at 37°C. This model was initially proposed for the study of the hemorrhagic activity of viperid venoms and showed a good correlation with the *in vivo* intradermal rodent assay (82). The model was then applied to the study of venom-induced lethality (83). The death of the embryo was assessed by observing the cessation of heart beats, followed by the submergence of the yolk sac membrane into the yolk (83). This model, however, cannot be applied for the study of neurotoxic venoms owing to the incipient development of neuromuscular junctions at this developmental stage in the chick embryo. The model was also used for assessing its correlation with *in vivo* toxicity, i.e. lethality, in the analysis of neutralization of nine venoms by antivenoms (84). A high correlation was found, suggesting the feasibility of using this system for evaluating antivenoms preclinical efficacy, except for neurotoxic venoms, for the reason indicated above. The model is more economic than those performed in mice and is also more convenient from the 3Rs perspective.

## Antivenomics

The application of -Omics technologies has had a high impact in the study of snake venoms, providing novel and relevant clues for understanding their evolution and composition in their ecological and medical contexts (85). In particular, the field of proteomics as applied to venoms, i.e. ‘venomics’ (86), has shed light on the complexity of these toxic secretions (87, 88). An application of the study of venom proteomes to the field of antivenoms is ‘antivenomics’, a translational venomics applied to the fine characterization of the ability of antivenoms to recognize different components in venoms.

Antivenomics methodologies have evolved through three ‘generations’. The baseline for antivenomic analysis is the proteomic characterization of venoms, with identification of the proteins and peptides after separation by reverse phase HPLC and one-dimension SDS-PAGE, and their quantification and classification in different protein families (86, 89). ‘First generation’ antivenomics was based on the incubation of venom and antivenom, followed by precipitation of immunocomplexes, and analysis of the supernatants containing venom proteins not recognized by antivenom antibodies (90). In ‘second generation’ antivenomics, the ability of antivenom antibodies to recognize venom components is assessed by affinity chromatography, whereby antibodies are bound to the chromatographic matrix and venom is passed through the columns. Hence, bound (reactive) and unbound (non-reactive) venom components are identified (91). The percentage of non-reactive venom component is then estimated based on the comparison between the areas under the peak of bound and unbound fractions, allowing a quantitative assessment of immune reactivity. In turn, ‘third generation’ antivenomics, which also uses affinity chromatography, enables the determination of the maximal binding capacity of antivenom antibodies for a particular toxin and also allows the quantification of the percentage of venom-specific antibodies in the whole antivenom (92).

Even though antivenomics is not a functional test in terms of neutralization of venom activities, it can shed valuable information for understanding the preclinical efficacy of antivenoms. The relative weight of venom components in the overall toxicity of a venom can be studied by determining the ‘toxicity score’ for each component, which takes into consideration the toxicity of each toxin and its relative abundance in the venom (24). Once the most relevant toxins in a venom are identified, the ability of antivenoms to recognize these components can be quantified through antivenomics, hence providing indirect evidence of efficacy of the antivenom.

It has been suggested that an antivenom is effective when it is able to immunocapture 20–25% of venom components (93), and the WHO guidelines for production, control and regulation of antivenoms indicate that an immunocapture capability of ≥25% of venom proteins generally correlates with a good outcome in the *in vivo* neutralization tests (5). Therefore, these guidelines recommend the use of antivenomics as a first screening test for the neutralizing ability of antivenoms, before moving to the *in vivo* tests (5). As indicated above, the application of antivenomics to the analysis of the ability of antivenoms to recognize the most toxic components in a venom, as identified by the toxicity score, further potentiates the analytical power of this *in vitro* method. This underscores the relevance of studying snake venoms from a functional ‘toxicovenomics’ approach, i.e., by combining venomics with characterization of toxicity profiles of individual venom fractions (88). **Table 1** summarizes the information available on *in vitro* assays that have shown correlation with *in vivo* tests in the assessment of antivenom neutralizing ability.

**Table 1:** Summary of the *in vitro* and *ex vivo* assays that have shown correlation with *in vivo* toxic activities of snake venoms in the assessment of the neutralizing ability of antivenoms.

Type of assay	Applications
Enzyme immunoassays (EIA)	Correlation with neutralization of lethality in some venoms and purified toxins
Passive hemagglutination	Correlation with neutralization of lethality in some venoms
Phospholipase A_2_ activity	Correlation with neutralization of lethality in some venoms
*In vitro* coagulant activity on plasma	Correlation with neutralization of lethality in some venoms. Correlation with defibrinogenating activity
Proteinase activity	Correlation with neutralization of hemorrhagic activity in some venoms
Cytotoxic activity on cells in culture	Possible correlation with neutralization of myotoxic and dermonecrotic activities of venoms*
Nerve-muscle preparations for assessing neuromuscular blockade	Possible correlation with neutralization of lethal and neurotoxic activities of venoms and isolated neurotoxins*
Binding to nicotinic acetyl choline receptors	Correlation with the neutralization of the lethal activity in venoms rich in post-synaptically acting α-neurotoxins
Antivenomics	Correlation with the neutralization of toxic components identified in venoms through proteomics and the toxicity score (toxicovenomics)

*In these cases, there have not been studies correlating observations in vitro and in vivo on the neutralization of these toxic activities; however, based on the mechanism of action of myotoxins, cytotoxins and neurotoxins, such correlation is highly likely.

## Toward Refining the Mouse Lethality Test

### The Introduction of Prophylactic Analgesia

Animal tests to assess venom toxicity and neutralization by antivenoms, particularly the mouse lethality assay, are associated with pain and distress, which may last for prolonged time intervals, as has been shown for crude venoms (94), and purified myotoxic PLA_2_s (95) and hemorrhagic SVMPs (96). The algogenic effect of venoms is due to the action of venom peptides and proteins that directly activate nociceptive (pain sensing) neural pathways, as well as by the action of endogenous inflammatory mediators released in tissues as a consequence of venom actions, which stimulate nociceptive receptors in neurons (94, 97). Despite the evident suffering induced in laboratory animals when assessing venom toxicity and neutralization by antivenoms, the scientific community in Toxinology, as well as antivenom manufacturers, have been slow at introducing interventions aimed at refining these tests with the use of analgesia. One reason might be the possibility that analgesia affects the results of the tests, although this assumption has not received experimental support. Hence, it is time to consider the routine use of precautionary analgesia in these tests, along the lines indicated by the WHO (5).

The analgesics such as buprenorphine (98), morphine and tramadol (99, 100) have been shown to be effective analgesics when used in experiments involving venoms that cause local tissue damage and death. No differences in the extent of local hemorrhage, edema and myonecrosis induced by venom of *Bothrops asper* in mice were observed in mice pre-treated with morphine and tramadol, as compared to controls not receiving analgesia (99). The analgesic effect of these drugs can be readily evaluated by using the Mouse Grimace Scale (MGS) (101) and the mouse exploratory activity (102), which enable the quantification of pain. It was shown that morphine and tramadol are effective in reducing pain in several models of envenoming by the venom of *B. asper* (100). Likewise, the use of tramadol did not alter the results of the estimation of antivenom potency in the case of *B. asper* venom and a polyspecific antivenom (37). It is necessary to expand these observations to other venoms to assess whether similar results are obtained. In that case, the routine use of analgesia should be promoted in research and quality control laboratories.

The duration of the action of these analgesics in mice must be considered. It has been estimated that it is between 2 and 3 h for morphine (103, 104) and up to 6 h for tramadol (105), whereas the action of buprenorphine in the rat lasts for 6–12 h (106). Hence, in experiments to assess lethality and its neutralization, which usually last for 24 h, there is a need of subsequent administrations of the analgesic. In the case of neurotoxic venoms, it is likely that opioid analgesics, such as the ones described, affect the outcome of the test. In these cases, the use of milder analgesics, such as paracetamol, could be considered.

### The Modification of the Protocol for the Lethality Test

The routine methods to estimate the LD_50_ of venoms and the ED_50_ of antivenoms usually last 24 or 48 h, depending on the route of injection (5, 6). Such prolonged time intervals involve much pain and distress in mice. Consequently, efforts are being carried out to make these tests less distressful. It is recommended that, before the assessment of venom LD_50_ or antivenom ED_50_, a range-finding test is done, in which only one mouse per venom or venom/antivenom level is used. In this way, the range of doses to be used in a complete experiment, which usually works with five to seix mice per group, can be selected without having to sacrifice too many mice (5). When the i.p. route is used in these tests, a 48 h observation period is established (5, 6). However, our unpublished observations at Instituto Clodomiro Picado reveal that the number of mice dead at 24 h is the same as at 48 h, hence not justifying observations beyond 24 h.

A more drastic shift in the protocol to assess venom LD_50_ and antivenom ED_50_ uses a maximum observation period of 8 h [see, for example, Barber et al. (107)]. In this methodology, envenomed animals are observed at regular time intervals, e.g., every hour, and the severity of envenoming is graded according to a pre-established set of parameters. Animals that are severely affected at any time interval, i.e., are moribund, are euthanized, and all animals surviving at the end of the 8-h observation period are also euthanized. This modification of the classical methodology reduces the extent of animal suffering, although it may affect the precision of the results, as it has been observed that mice that appear moribund may then recover. A balance needs to be made between the need to refine the lethality test and the need to ensure the robustness of the test for assessing antivenom efficacy. This urges the development of studies to assess the correlation between the results of these improved protocols and those of classical protocols.

## Concluding Remarks

**Figure 3:**
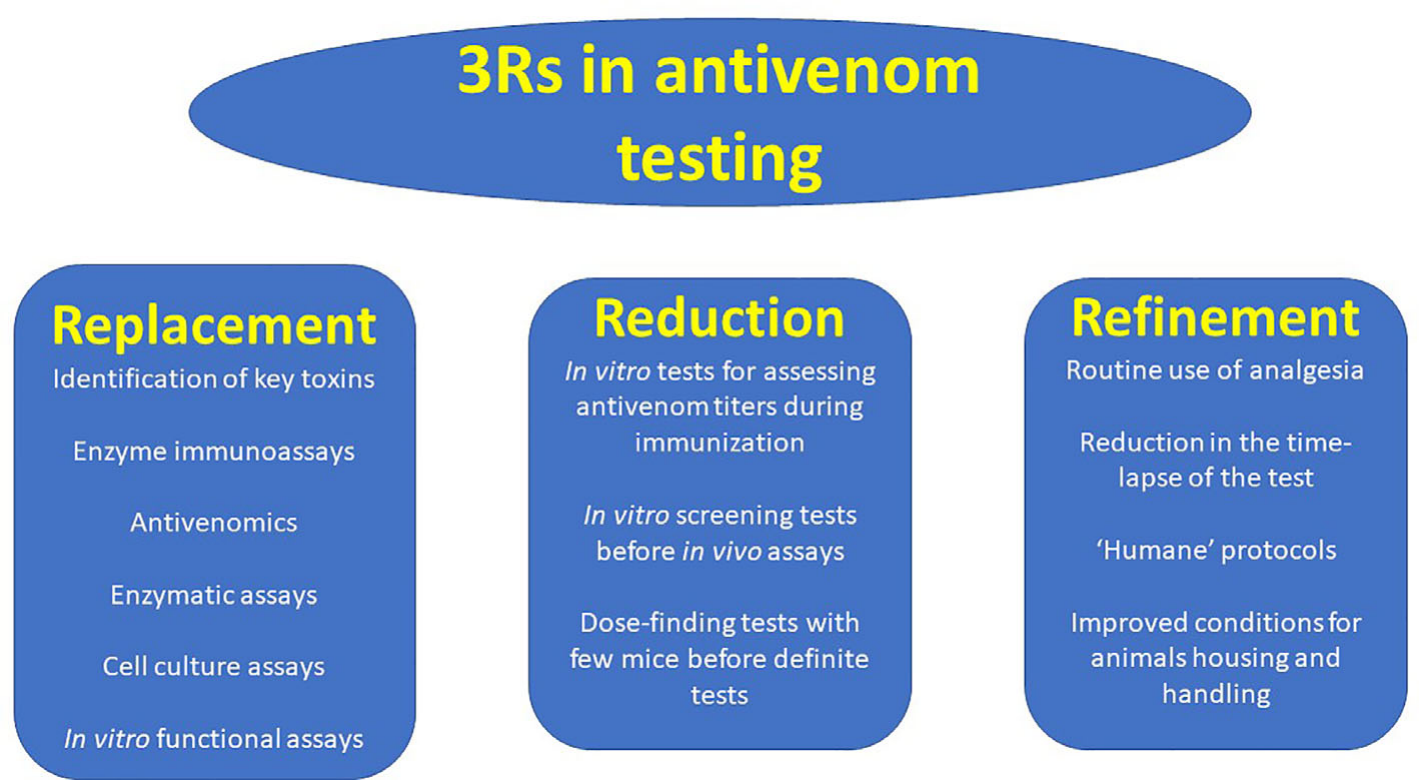
The 3Rs principles, as applied to the evaluation of the neutralizing ability of antivenoms. The search for Replacement, Reduction and Refinement (3Rs) should be actively pursued in the field of antivenom potency testing. Some examples of the implementation of these principles in antivenom testing are shown.

There is an urgent need to develop *in vitro* assays that correlate with *in vivo* toxicity tests in the study of venoms and in the assessment of the neutralizing ability of antivenoms, along with the 3Rs paradigm (**Figure 3**). This goal must be strengthened by research funding agencies and agendas, regulatory agencies and diverse stakeholders related to antivenom development, manufacture and quality control. This is a challenging task owing to the great complexity of the composition and mechanisms of action of venoms. A research-based, case by case analysis is needed in order to determine which is the most appropriate *in vitro* assay for each venom-antivenom system, providing the highest correlation with *in vivo* toxic activities, particularly lethality.

The best way to proceed along this line is to harness the growing body of information emerging from the study of venom toxicology and composition, which allows the identification of the most relevant toxic activities and toxins in each venom. This will facilitate the development of immunochemical or *in vitro* functional tests, enzymatic or otherwise, in substitution of animal-based assays. In turn, this calls for a closer collaboration between researchers in the biochemistry and pharmacology of venoms and toxins with professionals and technicians in antivenom production and quality control laboratories. Likewise, the regular use of analgesia in toxicity tests should be actively promoted in toxinological research and antivenom manufacture. It is expected that such initiatives will lead, in the short term, to a significant reduction in the number of animals used in research and antivenom development and potency evaluation, as well as in the suffering inflicted to those animals in the *in vivo* assays.

## Data Availability Statement

Inquiries on the sources of information used in this review can be directed to the corresponding author.

## Author Contributions

JG prepared the first version of this manuscript. MVa, AS, MH, MVi, GS, AS, CH, and GL revised and contributed to the content of the manuscript. All authors revised the final version of the work and agreed with its content. All authors contributed to the article and approved the submitted version.

## Funding

This work was supported by Vicerrectoría de Investigación, Universidad de Costa Rica.

## Conflict of Interest

The authors declare that the research was conducted in the absence of any commercial or financial relationships that could be construed as a potential conflict of interest.
